# Stunting and Physical Fitness. The Peruvian Health and Optimist Growth Study

**DOI:** 10.3390/ijerph17103440

**Published:** 2020-05-15

**Authors:** Carla Santos, Alcibíades Bustamante, Olga Vasconcelos, Sara Pereira, Rui Garganta, Go Tani, Donald Hedeker, Peter T. Katzmarzyk, José Maia

**Affiliations:** 1 CIFI2D, Faculty of Sport, University of Porto, 4200-450 Porto, Portugal; olgav@fade.up.pt (O.V.); sara.s.p@hotmail.com (S.P.); ruigarg@fade.up.pt (R.G.); jmaia@fade.up.pt (J.M.); 2 School of Physical Education and Sports, National University of Education Enrique Guzmán y Valle, 60637 La Cantuta, Lurigancho-Chosica 15472, Peru; huanta2609@yahoo.es; 3 School of Physical Education and Sports, University of São Paulo, São Paulo 05508-030, Brazil; gotani@usp.br; 4 Department of Public Health Sciences, University of Chicago, Chicago, IL 60637, USA; dhedeker@health.bsd.uchicago.edu; 5 Pennington Biomedical Research Center, Louisiana State University, Baton Rouge, LA 70808, USA; peter.katzmarzyk@pbrc.edu

**Keywords:** children and adolescents, growth, stunting, physical fitness

## Abstract

Stunting, defined as linear growth retardation, is a serious public health problem in developing countries. We aimed to (1) describe the prevalence of stunting in Peruvian youth living in three geographical regions, and to (2) determine height and physical fitness (PF) differences between stunted and normal-growth children across age and sex. We sampled 7918 subjects (7074 normal-growth and 844 stunted), aged 6–15 year, from sea-level, Amazon and high-altitude regions of Peru. PF was assessed with standardized tests, and stunting was computed following World Health Organization (WHO) standards. A two-factor analysis of variance (ANOVA) model was used. Results showed that stunting prevalence increased with age (from 6% at 6 year to 18.4% at 15 year in girls, and 9.3% at 6 year to 16.4% at 15 year in boys); was higher in boys (12.3%) than in girls (9.3%), and was higher in the Amazon region (25.3%), followed by high-altitude (24.3%) and sea-level (8.1%). Stunting had a negative overall impact on girls’ and boys’ statures. Further, the age-by-stunting interactions were statistically significant for both sexes, and significant differences in height varied to some degree across age. Stunted children performed worse in handgrip and standing long jump, but outperformed their normal-growth peers in shuttle-run (only boys), and in 12 min run. Further, significant differences in the age-by-stunting interaction occurred in all PF tests, varying to some degree across age. In conclusion, stunting significantly affects Peruvian youth’s PF levels, and this influence is sex-, age- and PF test-specific.

## 1. Introduction

Stunting, i.e., impaired growth and development mostly resulting from poor nutrition, is a serious public health problem during childhood and adolescence in both low and middle income countries [1]. Across the lifespan, stunting has been associated with increased risk of morbidity and mortality, as well as increased risk of obesity [2]. It also expresses itself in physical, cognitive and motor development delays, with multiple consequences in adulthood [2]. Consequently, stunting tends to lead to lower economic productivity and constrained social functioning, being a direct impediment to achieving sustainable developmental goals, such as achieving gender equality, as well as reducing inequality within and between countries [3].

A secular trend investigation [4] using available data from 1990 to 2015 showed that stunting affects 171 million children worldwide, and that most of those affected live in developing countries. Further, it was also reported that childhood stunting decreased from 39% in 1990 to 24.1% in 2015, and that different patterns occur in developing and developed countries. For example, in developed countries, stunting has been stable at 6% since 1990 and is expected to remain at this level, whereas in developing countries a decrease from 44.4% in 1990 to 26.2% in 2015 was found, and it is also expected to decrease further to 23.7% by 2020.

Although in Latin America the stunting prevalence (11.6%, or 6 million in 2015) is lower than in Africa (37.6%) and Asia (22.9%) [4], it is considered a very serious health problem, and Peru is no exception. For example, Urke et al. [5], using data from Peruvian Demographic and Health Surveys, revealed that in 2011, the prevalence of stunting increased with increasing age in both sexes: from 20.5% at ages 6–11 year to 25.4% at ages 12–23 year in boys, and from 14% at ages 6–11 year to 21.1% at ages 12–23 year in girls; additionally, the prevalence was somewhat higher in boys (19.8%) than in girls (18.8%). Importantly, there are geographic differences in stunting in Peru: children living on the coast (8.3% of boys and 6.8% of girls) are less stunted than those from the Andean (29.7% of boys and 29.6% of girls) and Amazon (26.5% of boys and 23.5% of girls) regions [5]. Disparities in Peruvian life conditions, characterized by economic, educational, nutritional, and health resources inequalities, still exist today, and may help explain these differences in stunting prevalence. 

It has been consistently reported that stunting adversely affects health markers, like loss of physical growth potential, reduced neural development and cognitive function, as well as an elevated risk of chronic disease in adulthood [2,6]. However, very few studies have investigated the impact of stunting on physical fitness (PF) among children and adolescents. Further, although it is well acknowledged that PF is an important health marker [7], this information is not available for Peruvian youth. It has also been shown that adequate PF levels during childhood and adolescence are linked to a higher cognitive development [8], academic performance [9], and a healthier cardiovascular profile during adulthood [10]. Moreover, in developing countries like Mozambique [11], Senegal [12], México [13] and Colombia [14], stunted children tend to have worse performance levels in a variety of PF tasks. This influence is apparently sex-, age- and PF test-specific. For example, Malina, Pena Reyes, Tan and Little [13] showed that stunted Mexican youth of both sexes, aged 6 to 13 year, significantly differ from their normal-growth peers in both handgrip strength and standing long jump, whereas Arsenault, Mora-Plazas, Forero, Lopez-Arana, Jáuregui, Baylin, Gordon and Villamor [14] reported that stunted Colombians, aged 5 to 12 year, scored significantly lower in the shuttle-run test in both sexes, but in the standing long jump test this relationship was only significant in boys. Similar results were described in Mozambican [15], Macedonian [16] and South African [17] children and adolescents. 

Bearing in mind James Tanner´s maxim that “growth is a mirror of the society” [18], we believe that the identification of Peruvian children’s impaired growth is of utmost importance in educational and public health terms. Further, as far as we know, no studies have scrutinized how Peruvians’ PF levels, as suitable health markers, depend on their stunting condition. Hence, we intend to answer the following questions: (1) What is the prevalence of stunting in Peruvian youth residing in distinct geographical areas? (2) Are there systematic differences in height and PF levels between stunted and normal-growth youth? (3) Is the impact of stunting in physical fitness levels consistent across age? (4) Is this difference sex-, age- and physical fitness test-specific?

## 2. Materials and Methods 

### 2.1. Design and Participants

The sample of this study is part of a larger project titled *“The Peruvian Health and Optimist Growth Study”* [19], carried out between 2009 and 2010, and in which participants were randomly sampled from schools in three geographical areas in the central region of Peru: sea-level (Barranco—58 m), Amazon region (La Merced and San Ramon—751 m), and high-altitude (Junín—4107 m). We sampled 7918 children and adolescents (4388 girls, aged 6–15 year), with complete data on PF tests. The percentage of missing data varied from 4% to 10% in PF tests, and did not significantly differ (*p* > 0.05) from those considered in the present paper. The sample only included children and adolescents with complete data on all variables, and who also were natives to their respective regions (i.e., no immigrants were identified). Information about the participant’s birth place and current place of residence or address was collected and cross-checked with their identity cards. All assessments were carried out in Barranco between November and December (2009); in April and July (2010) in La Merced and between May and August in San Ramon; and between September and October of 2009 in Junín. Formal permissions from school governmental bodies and written informed consent from parents/legal guardians were obtained. The project was approved by the Ethics Committee of the National University of Education Enrique Guzmán y Valle (UNE EGyV).

### 2.2. Measurements and Tests

#### 2.2.1. Anthropometry

Height (cm) and sitting height (cm) were measured to the nearest 0.1 cm according to standardized procedures [20], using a portable stadiometer (Personal Caprice Sanny^®^, Model ES-2060, São Bernardo do Campo, SP, Brazil) holding the child´s head in the Frankfurt plane. Body weight was measured to the nearest 0.1 kg with a digital scale (Pesacon, Model IP68).

#### 2.2.2. Biological Maturation

Biological maturation was assessed with the maturity offset [21], which estimates time before or after age-at-peak height velocity (APHV). A sex-specific formula based on age, sex, height, sitting height, and weight was used. A positive (+) maturity offset represents the number of estimated years a child is beyond APHV; a negative (–) value represents the number of estimated years a child is before APHV, whereas a zero value indicates that a child is experiencing his/her APHV.

#### 2.2.3. Physical Fitness

PF was assessed with a variety of measures. Handgrip strength (kgf—marker of static strength) was measured using a hand dynamometer (Takei Hand Grip Dynamometer^®^, Takei Scientific Instruments Co., Ltd, Nigata, Japan)—all participants gripped the dynamometer with maximum force for 5 to 10 s. Standing long jump (centimeters—marker of lower body explosive strength) was assessed via a maximum jump distance recorded from two trials. A shuttle-run (seconds—marker of agility) was performed where children completed five cycles (round-trip) at maximum speed between two lines separated by five meters. A 12 min run (meters—marker of aerobic fitness) was performed in a previously delimited field where schoolchildren ran/walked the maximum possible distance in 12 min. The first three tests were from EUROFIT [22], and the 12 min run from The American Alliance for Health, Physical Education and Recreation (AAHPERD) [23].

#### 2.2.4. Stunting

Stunting was computed using references for children and adolescents 5 to 19 year of age as advocated by Onis et al. [24]. Thus, subjects were classified as stunted (height-for-age Z score < −2 Standard Deviation (SD) using age- and sex-specific reference heights according to the WHO standards [25], according to the STATA software syntax provided by the WHO [26]. 

### 2.3. Data Quality Control

Data quality control was assured by a series of steps. Firstly, all team members were trained on the technical procedures of body measurements, and the PF tests and assessment protocols were strictly followed by all team members and supervised by the principal investigator. Secondly, a pilot study was conducted to assess the quality of data collection. Thirdly, a random sample was retested. The inter-observer technical errors of the measurement were 0.2 cm for height, 0.1 cm for sitting height, and 0.1 kg for weight. ANOVA-based intraclass correlation coefficient for PF tests ranged from 0.79 (12 min run) to 0.85 (standing long jump), respectively. Finally, data cleaning was performed, using IBM-SPSS v26, to control for punching errors in data entry, the presence of outliers, as well as normality checks in the distributions of all variables.

### 2.4. Statistical Procedures

Basic descriptive statistics [means, standard deviations (SD), and percentages (%)] were computed. Normality checks for the distributions of height and PF were examined using the D’Agostino et al. [27] test implemented in STATA software, and no violations were encountered. A two-factor ANOVA model, adjusted for geographical location, was used to test for height and PF differences across age and stunting condition (main effects), as well as an interaction of age-by-stunting within each sex, and the partial eta squared (η^2^) was used as a measure of effect size. Further, differences between conditions (stunted versus non-stunted) for each age group within each sex were tested with the margins procedure implemented in STATA v14 software, which was also the software used in all analysis. The significance level was set at 5%.

## 3. Results

Table 1 provides descriptive information about the prevalence of stunting by age, sex and geographical area of residence. In the total sample, the prevalence of stunting was 11%. The prevalence seems to increase with increasing age in both sexes, from 6% at 6 year to 18.4% at 15 year in girls, and from 9.3% at 6 year to 16.4% at 15 year in boys. Further, it is higher in boys (12.3%) than in girls (9.3%), and was higher among children and adolescents living in the Amazon region (25.3%), followed by high-altitude (24.3%) and sea-level (8.1%).

**Figure 1:**
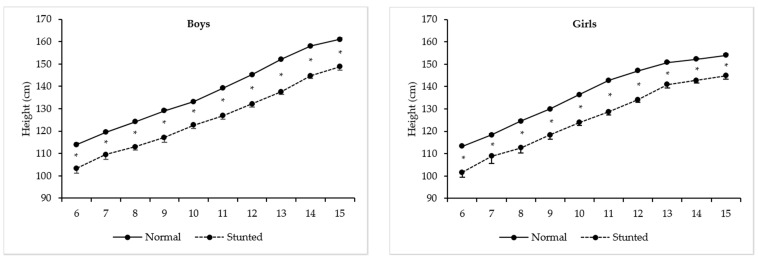
Mean levels for height between stunted and normal-growth Peruvian girls and boys (* marks statistically significant differences; 95% Confidence intervals).

Table 2 and Figure 1 show the two-factor ANOVA model results for differences in height between stunted and normal-growth children in both sexes, respectively. Stunting means are statistically significant with negative overall impacts in girls´ [F(1, 4366) = 1277.20, *p* < 0.01, η^2^ = 0.23], as well as in boys´ [F(1, 3508) = 1716.15, *p* < 0.01, η^2^ = 0.33] statures. The age-by-stunting interactions proved to be statistically significant for girls [F(9, 4366) = 4.50, *p* < 0.01, η^2^ = 0.01] and boys [F(9, 3508) = 3.03, *p* < 0.01, η^2^ = 0.01]. Marked significant differences in height varied to some degree across the ages, starting at 6 year through 15 year [in girls, the lowest difference was found at 15 year (9.1 cm), and the highest at 11 year (14.0 cm); in boys, the lowest difference was found at 7 year (10.0 cm), and the highest at 13 year (14.5 cm)].

**Figure 2:**
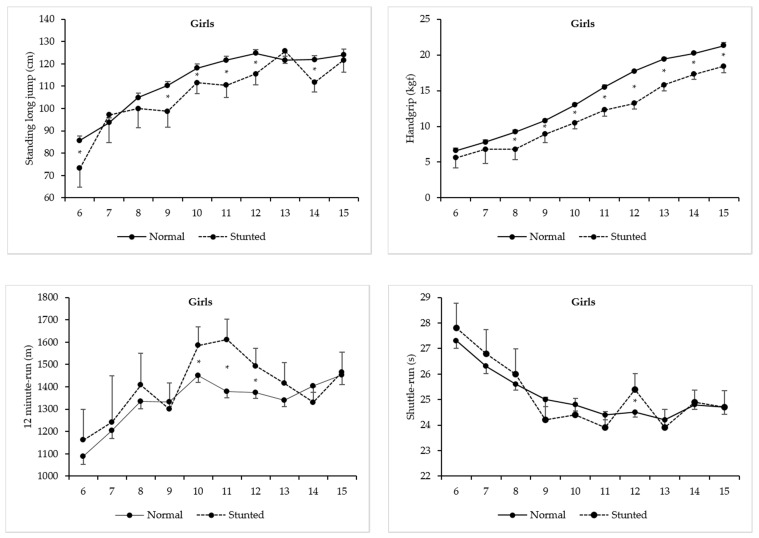
Mean levels for each physical fitness test between stunted and normal-growth Peruvian girls (* marks statistical significant differences; 95% Confidence intervals).

Table 3 and Figure 2 show two-factor ANOVA model results for each PF test between stunted and normal-growth girls. With increasing age, girls were significantly stronger in handgrip [F(9, 4366) = 294.43, *p* < 0.01, η^2^ = 0.38], with more explosive lower body strength [F(9, 4366) = 48.66, *p* < 0.01, η^2^ = 0.09], as well as more agile [F( = 9, 4366) = 16.72, *p* < 0.01, η^2^ = 0.03], and with higher aerobic fitness [F(9, 4366) = 14.57, *p* < 0.01, η^2^ = 0.03]. Stunting is statistically significant, with negative impacts in handgrip [F(1, 4366) = 181.07, *p* < 0.01, η^2^ = 0.04] and standing long jump [F(1, 4366) = 26.57, *p* < 0.01, η^2^ = 0.01], but with positive impact in 12 min run test [F(1, 4366) = 11.00, *p* < 0.01, η^2^ = 0.003]. Further, it was not statistically significant for shuttle-run [F(1, 4366) = 2.13, *p* = 0.14, η^2^ = 0.000]. The age-by-stunting interactions proved to be statistically significant for all PF tests [handgrip: F(9, 4366) = 3.36, *p* < 0.01, η^2^ = 0.01; standing long jump: F(9, 4366) = 2.99, *p* < 0.01, η^2^ = 0.01; shuttle-run: F(9, 4366) = 2.48, *p* < 0.01, η^2^ = 0.01; and 12 min run: F(9, 4366) = 3.56, *p* < 0.01, η^2^ = 0.01]. Marked significant differences in the interaction occurred in all PF tests, though varied to some degree across the ages. In handgrip, significant differences occurred from 8 to 15 year [the lowest difference was found at 9 year (1.9 kgf), and the highest at 12 year (4.5 kgf)]. In standing long jump, significant differences occurred at 6, 9, 10, 11, 12 and 14 year [the lowest difference was found at 10 year (6.6 cm), and the highest at 11 year (11.2 cm)], while in 12 min run, significant differences occurred at 10 year till 12 year [the lowest difference was found at 12 year (−117.3 m), and the highest at 11 year (−230.8 m)]. Finally, in shuttle-run, significant differences in the age-by-stunting interaction only occurred at 12 year (−0.9 s).

**Figure 3:**
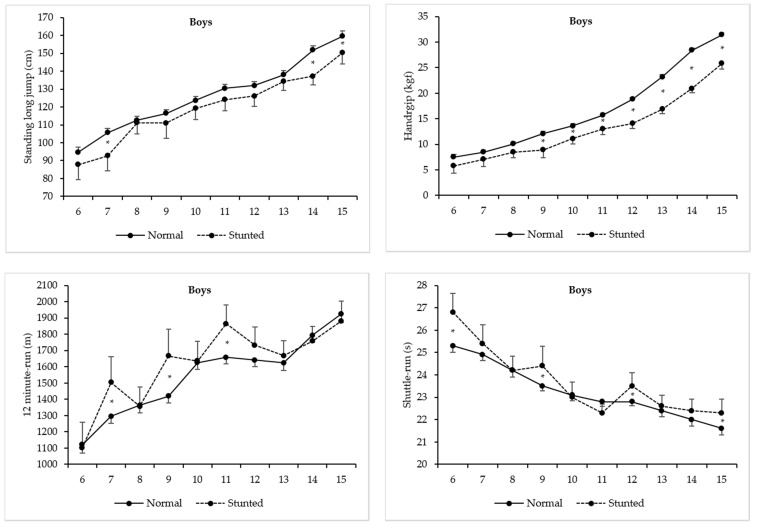
Mean levels for each physical fitness test between stunted and normal-growth Peruvian boys (* marks statistical significant differences; 95% Confidence intervals).

Table 4 and Figure 3 show two-factor ANOVA model results comparing PF levels between stunted and normal-growth boys. Older boys were significantly stronger in handgrip [F(9, 3508) = 571.24, *p* < 0.01, η^2^ = 0.59], had more explosive lower body strength [F(9, 3508) = 109.14, *p* < 0.01, η^2^ = 0.22], were more agile [F(9, 3508) = 44.24, *p* < 0.01, η^2^ = 0.10], and showed higher aerobic fitness [F(9, 3508) = 40.36, *p* < 0.01, η^2^ = 0.09]. Stunting is statistically significant with negative impacts in handgrip [F(1, 3508) = 351.53, *p* < 0.01, η^2^ = 0.09] and standing long jump [F(1, 3508) = 37.81, *p* < 0.01, η^2^ = 0.01], but with positive effects in shuttle-run [F(1, 3508) = 11.51, *p* < 0.01, η^2^ = 0.003] and 12 min run test [F(1, 3508) = 10.14, *p* < 0.01, η^2^ = 0.003]. The age-by-stunting interactions were statistically significant for all PF tests [handgrip: F(9, 3508) = 14.99, *p* < 0.01, η^2^ = 0.04; standing long jump: F(9, 3508) = 2.10, *p* < 0.01, η^2^ = 0.01; shuttle-run: F(9, 3508) = 2.43, *p* < 0.01, η^2^ = 0.01; and 12 min run: F(9, 3508) = 2.43, *p* < 0.01, η^2^ = 0.01]. As with girls, marked significant differences in the interaction also occurred in all PF tests, though varied to some degree across the ages. In handgrip, significant differences occurred from 9 to 15 y [the lowest difference was found at 10 y (2.5 kgf), and the highest at 14 y (7.5 kgf)]. In standing long jump, significant differences occurred at 7, 14 and 15 y [the lowest difference was found at 15 y (9.2 cm), and the highest at 14 y (14.7 cm)], while in shuttle-run, significant differences occurred at 6, 9, 12 and 15 year [the lowest differences was found at 12 (−0.7 s) and 15 year (−0.7 s), and the highest at 6 y (−1.5 s). Finally, in 12 min run, significant differences occurred at 7, 9, and 11 year [the lowest difference was found at 11 year (−205.4 m), and the highest at 9 year (−248.0 m)]. 

## 4. Discussion

We found a stunting prevalence of 11% in Peruvian children and adolescents aged 6 to 15 year, with significant increases with increasing age in both sexes, as well as a higher prevalence in boys than in girls. Similar trends were also reported by Urke, Mittelmark and Valdivia [5] in 2011, in a study conducted on Peruvian youth, where stunting intensifies with age in both sexes, from 20.5% at age range of 6–11 year to 25.4% at 12–23 year in boys, and from 14% at 6–11 y to 21.1% at 12–23 year in girls; and boys (19.8%) were also more stunted than girls (18.8%). It is possible that the presence of sex-differences in these cases of prevalence may be related to cultural dissimilarities existing in the distribution of, and access to, nutritional and health resources. For example, in sub-Saharan Africa, Wamani et al. [29] stated that male children under five years of age were more likely to become stunted because they are more vulnerable to health inequalities than their female peers. Further, it has also been reported that in societies where boys are favored over girls, the reverse effect is often found, reflecting a higher female vulnerability [30]. Of significance is that in Peru, stunting decreased across time, since later-born children also presented lower prevalence. This might suggest an improvement in Peruvian children’s quality of life across time (namely, better feeding practices, enhanced household sanitation practices, maternal nutrition, etc.) [31,32].

We also observed geographic variations in stunting prevalence favoring those living at sea-level, followed by high-altitude and Amazon region peers. Urke, Mittelmark and Valdivia [5] also described lower stunting rates among Peruvian children living at the coast (8.3% of boys and 6.8% of girls) than those from the Andean (29.7% of boys and 29.6% of girls) and Amazon (26.5% of boys and 23.5% of girls) regions. These geographical differences may be explained by higher poverty observed at moderate and high altitudes, where the market penetration is less common, and the main livelihoods are predominantly stockbreeding and non-diversified agriculture, reflecting a poor quality of the families’ diet, with a restricted nutritional intake, which, consequently, affects children and adolescent’s health and growth status [33,34]. The stunting phenomenon may be related to environmental stressors, since children living at high-altitude have to deal with hypoxic stress, as well as an adverse mountain climate, which could considerably affect their linear growth patterns [35,36,37]. Although the present study was carried out about 10 years ago, socio-geographical differences have remained among the three areas, in terms of economic, educational, nutritional and health resource inequalities. Recent data by the World Bank in Peru [38] showed that the country had a sustained economic growth between 2002 and 2013, with an average gross domestic product (GDP) growth rate of 6.1% per year, generating a high-growth and low-inflation scenario. However, sustained macroeconomic growth at this stage was not reflected in the microeconomics of most Peruvian families. The expansion of the economy slowed to an average of 3.1% per year between 2014 and 2019, mainly due to changes in international commodity prices, with a temporary fall in private investment, lower tax revenues and a slowdown in consumption. Thus, as our sample corresponds to 76.7% of public schoolchildren, this indicates that in most Peruvian families, no substantial and systematic changes in socioeconomic conditions occurred in the last decade. 

As expected, with increasing age, Peruvian children and adolescents become significantly taller, but stunting has a negative overall effect on their statures. This is completely expected, as the definition of stunting is a low height-for-age. We found that children from both sexes and all ages experienced stunting. That said, the highest differences occurred between 9 and 12 year in girls and between 12 and 14 y in boys. This timing apparently coincides with the growth spurt in height, which may help explain the highest differences in stature in this specific age group. We have recently reported [31] that peak height velocity occurred approximately at 9.68 y of age in Peruvian girls, and approximately two years later in boys (≅ 12.61 year) living at different altitudes (sea-level, Amazon region and high-altitude). A similar trend was also described by Cossio-Bolanos, Campos, Andruske, Flores, Luarte-Rocha, Olivares, Garcia-Rubio and de Arruda [34], where Peruvian girls living at moderate altitude (~2.328 m) experienced their peak high velocity (PHV) at 12.7 year and boys at 15.2 year, which coincides with the moment of greatest delay in their linear growth (from 12 to 14 year in girls, and from 15 to 17 year in boys). It is possible that different sample sizes, different regions considered, as well as the mathematical model used, may be responsible for the differences in Peruvian boys and girls mean ages at PHV in these both studies. 

Another important finding was that stunting significantly affected PF levels, and this influence was sex-, age- and PF test-specific. In handgrip, normal-growth girls and boys outperformed their stunted peers, and significant differences occurred from childhood to adolescence. These results emphasize the consequences of reduced body size and muscle mass associated with stunting early in life. Stunted children were, on average, “deficient” in muscle tissue needed to generate force [13]. In turn, normal-growth children also seemed to be favored in standing long jump, with significant differences between sexes across age. Since this test requires children to rapidly change their center of mass and reach a maximum jumping distance, it is expected that those with shorter lower extremities are usually less capable to do so effectively, and the net result is their lower performance. Although reduced body size and muscle mass also influence jumping performance, stunted children are also expected to mature later, and in all likelihood, this affects their jumping patterns. Fundamental movement skills (running, kicking, throwing, hopping, skipping, etc.) are generally mature by about 6–8 year in normal-growth children, but in the standing long jump, the mature form is reached by 9–10 year [39]. Thus, later maturation, and perhaps motor coordination developmental delay linked to early growth “retardation”, may be additional factors explaining the poorer jumping performances of stunted Peruvians when compared with their normal-growth peers. These results were corroborated by Malina, Pena Reyes, Tan and Little [13], showing that stunted Mexican youth of both sexes, aged 6 to 13 year, significantly differ from their normal-growth peers in both handgrip strength and standing long jump, whereas Arsenault, Mora-Plazas, Forero, Lopez-Arana, Jáuregui, Baylin, Gordon and Villamor [14] reported that stunted Colombians aged 5 to 12 year scored significantly lower in the standing long jump, even though this relationship was only statistically significant in boys.

Our results also revealed that stunted boys and girls have an apparent mechanical advantage in PF tasks involving greater energy expenditure, spending less time to complete the shuttle-run test, as well as covering a greater distance in running/walking the 12 min test. One possible explanation may be linked to the fact that a greater body size also means a greater body weight, and consequently greater fat mass (mainly in girls), which may negatively affect children’s motor performance. This has been shown to be true in tasks that require agility, with rapid changes of direction [40], as well as tasks that require more cardiorespiratory capacity [40]. However, we found that significant differences only occurred within specific ages in shuttle-run (only at 12 year in girls and at 6, 9, 12 and 15 year in boys), and in 12 min run test (starting at 10 y till 12 year in girls, and at 7, 9 and 11 y in boys), which suggest that, in some ages, the stunting condition did not affect their motor performance. We think that Peruvians’ general improvements in agility and in cardiorespiratory capacity are associated with their body build and shape, as well as physiological characteristics resulting from adaptation to environmental conditions. It is also possible that demands of their daily chores and manifold physical activities may help explain these results [41]. Similarly, Tambalis et al. [42] found that thin Greek schoolchildren aged 4–17 year (considering thinness as related to holdup in somatic growth and development among children) performed better than normal weight peers in aerobic fitness test (multi-stage 20 m shuttle run test). Similarly, thin Mozambican children, aged 6–18 y, performed better in endurance tests, and equally in agility test, as compared to the normal weight group [15]. The same trend was found among Egyptian [43] and South African under-nourished children [17]. Differently, Nhantumbo, Ribeiro Maia, Dos Santos, Jani, Gudo, Katzmarzyk and Prista [11] reported that Mozambican stunted children performed less well in the one mile run test in both sexes, and in the 10 × 5 m agility run test, but only in girls. In sum, this issue remains controversial, requiring more studies to best explain the relationship between agility and cardiorespiratory PF components in stunted boys and girls.

The present study is not without limitations. First, the sample is not representative of the Peruvian population, which restricts the generalization of the results. However, we have a fairly large sample (7918 subjects), aged 6–15 year, covering an important time window in Peruvians’ growth and development. Second, no information was collected about children’s nutritional habits, as well as about the putative influence of family income and/or socioeconomic status. However, the financial costs associated with collecting data on 8000 families made this option unfeasible. Thirdly, the time of day of measurements was not the same for all children, given known geographical constraints, as well as school schedules. In fact, measuring thousands of children and adolescents from different socio-geographic areas at precisely the same time of the day, during their school-time, was impossible. However, of all studies consulted in this report, only one [43] mentioned this issue, but did not measure their subjects at the same time of the day. Despite these limitations, the strong point of this study is that it included a large sample of Peruvian children and adolescents from three different geographical locations, and provides relevant information about their impaired growth, as well as how their PF levels depend on their stunting condition.

## 5. Conclusions

In summary, stunting prevalence increased with age, was higher in boys than in girls, and was also higher in children and adolescents living in the Amazon region, followed by high-altitude and sea-level. Stunting had a negative overall impact on girls´ and boys´ statures. The age-by-stunting interactions proved to be statistically significant for both sexes, and significant differences in height varied to some degree across the ages. Stunted children performed worse in handgrip and standing long jump, but outperformed their normal-growth peers in shuttle-run (only boys), and in 12 min run. Further, significant differences in the age-by-stunting interaction occurred in all PF tests, varying to some degree across the ages. In conclusion, stunting significantly affects Peruvian youth’s PF levels, and this influence is sex-, age- and PF test-specific. Our results provide valuable information that should be considered, on the one hand, by Peruvian political authorities when designing more efficient strategies to combat the stunting problem, creating a sustained and equitable implementation of multisector interventions, with focus on the poorest regions of the country. On the other hand, Peruvian school principals, councils and Physical Education teachers have to bear in mind the significance of these results when designing local intervention programs to help stunted children to increase their PF levels. 

## Figures and Tables

**Table 1:** Sample size and prevalence of stunting (%) by age, sex and geographical area of residence.

	Age(y)^†^	Sea-Level (Barranco)		Amazon region (La Merced and San Ramon)		High-Altitude (Junín)		All Areas	
**Girls**		Normal	Stunted (%)	Normal	Stunted (%)	Normal	Stunted (%)	Normal	Stunted (%)
	6	102	2 (1.8)	159	18 (10.2)	50	0 (0.0)	311	20 (6.0)
	7	74	0 (0.0)	155	5 (3.1)	78	4 (4.9)	307	9 (2.8)
	8	82	5 (5.7)	224	14 (5.9)	70	0 (0.0)	376	19 (4.8)
	9	121	5 (4.0)	201	15 (6.9)	85	7 (7.6)	407	27 (6.2)
	10	95	4 (4.0)	244	44 (15.3)	85	9 (9.6)	424	57 (11.9)
	11	141	2 (1.4)	214	28 (11.6)	97	15 (13.4)	452	45 (9.1)
	12	115	0 (0.0)	280	40 (12.5)	171	20 (10.5)	566	60 (9.6)
	13	88	1 (1.1)	268	23 (7.9)	91	21 (18.8)	447	45 (9.1)
	14	138	11 (7.4)	210	39 (15.7)	122	29 (19.2)	470	79 (14.4)
	15	78	13 (14.3)	69	24 (25.8)	71	12 (14.5)	218	49 (18.4)
	Total	1034	43 (4.0)	2024	250 (11.0)	920	117 (11.3)	3978	410 (9.3)
		Normal	Stunted (%)	Normal	Stunted (%)	Normal	Stunted (%)	Normal	Stunted (%)
	6	44	0 (0.0)	133	21 (13.6)	57	3 (5.0)	234	24 (9.3)
	7	28	0 (0.0)	218	23 (9.5)	59	1 (1.7)	305	24 (7.3)
	8	36	0 (0.0)	182	37 (16.9)	80	4 (4.8)	298	41 (12.1)
	9	42	1 (2.3)	231	16 (6.5)	105	5 (4.5)	378	22 (5.5)
**Boys**	10	39	3 (7.1)	217	30 (12.1)	96	9 (8.6)	352	42 (10.7)
	11	78	4 (4.9)	219	30 (12.0)	69	11 (13.8)	366	45 (10.9)
	12	99	3 (2.9)	156	36 (18.8)	120	9 (7.0)	375	48 (11.3)
	13	32	0 (0.0)	138	39 (22.0)	103	33 (24.3)	273	72 (20.9)
	14	71	2 (2.7)	128	34 (21.0)	107	39 (26.7)	306	75 (19.7)
	15	75	10 (11.8)	78	18 (18.8)	56	13 (18.8)	209	41 (16.4)
	Total	544	23 (4.1)	1700	284 (14.3)	852	127 (13.0)	3096	434 (12.3)

Note: ^†^ Age was used as decimals of a year, and children aged 5.5 to 6.49 year were designated as 6, children aged 6.50 to 7.49 year as 7, and so on [28].

**Table 2:** Mean differences (Mean ± SD, F tests and Dif) and two-factor analysis of variance (ANOVA) model results for height between stunted and normal-growth children of both sexes.

Height (cm)								
	Girls				Boys			
Age (y)	Normal	Stunted	F	Dif	Normal	Stunted	F	Dif
6	113.3 ± 0.3	101.6 ± 1.1	101.23	11.7 *	114.0 ± 0.3	103.4 ± 1.1	91.09	10.6 *
7	118.4 ± 0.3	108.9 ± 1.7	31.66	9.5 *	119.5 ± 0.3	109.5 ± 1.1	81.73	10.0 *
8	124.5 ± 0.3	112.6 ± 1.2	101.18	11.9 *	124.1 ± 0.3	113.0 ± 0.8	164.11	11.1 *
9	130.0 ± 0.2	118.4 ± 1.0	134.30	11.6 *	129.1 ± 0.3	117.1 ± 1.1	111.63	12.0 *
10	136.4 ± 0.2	123.9 ± 0.7	309.88	12.5 *	133.2 ± 0.3	122.7 ± 0.8	154.13	10.5 *
11	142.7 ± 0.2	128.7 ± 0.7	313.31	14.0 *	139.2 ± 0.3	126.9 ± 0.8	222.67	12.3 *
12	147.0 ± 0.2	134.1 ± 0.7	356.82	12.9 *	145.2 ± 0.3	132.1 ± 0.7	270.17	13.1 *
13	150.7 ± 0.2	140.9 ± 0.8	156.24	9.8 *	152.1 ± 0.3	137.6 ± 0.6	448.21	14.5 *
14	152.2 ± 0.2	142.7 ± 0.6	240.42	9.5 *	158.0 ± 0.3	144.7 ± 0.6	392.70	13.3 *
15	153.9 ± 0.3	144.8 ± 0.7	132.72	9.1 *	161.0 ± 0.4	148.8 ± 0.8	188.80	12.2 *
**Two-Factor ANOVA Model Results**								
**Age**	F(9, 4366) = 894.06, *p* < 0.01, η^2^ = 0.65				F(9, 3508) = 1206.54, *p* < 0.01, η^2^ = 0.76			
**Stunted**	F(1, 4366) = 1277.20, *p* < 0.01, η^2^ = 0.23				F(9, 3508) = 1206.54, *p* < 0.01, η^2^ = 0.76			
**Age by Stunted**	F (9, 4366) = 4.50, *p* < 0.01, η^2^ = 0.01				F(9, 3508) = 3.03, *p* < 0.01, η^2^ = 0.01			

Note: * *p* < 0.05.

**Table 3:** Mean differences (Mean ± SD, F tests and Dif) and two-factor ANOVA model results for each physical fitness test between stunted and normal-growth girls.

Age (y)	Handgrip (kgf)				Standing Long Jump (cm)				Shuttle-Run (s)				12 min run (m)			
	Normal	Stunted	F	Dif	Normal	Stunted	F	Dif	Normal	Stunted	F	Dif	Normal	Stunted	F	Dif
6	6.6 ± 0.2	5.6 ± 0.7	2.10	1.0	85.7 ± 1.1	73.2 ± 4.3	8.10	12.5 *	27.3 ± 0.1	27.8 ± 0.5	0.85	−0.5	1087.9 ± 17.9	1161.4 ± 70.5	1.02	−73.5
7	7.8 ± 0.2	6.8 ± 1.0	0.83	1.0	93.7 ± 1.1	97.1 ± 6.3	0.27	−3.4	26.3 ± 0.1	26.8 ± 0.4	6.28	−0.5	1203.8 ± 18.0	1242.2 ± 105.0	0.13	−38.4
8	9.2 ± 0.2	6.8 ± 0.7	11.39	2.4 *	104.9 ± 1.0	99.9 ± 4.4	1.28	5.0	25.6 ± 0.1	26.0 ± 0.5	0.38	−0.4	1334.3 ± 16.3	1408.4 ± 72.3	1.00	−74.1
9	10.8 ± 0.2	8.9 ± 0.6	9.51	1.9 *	110.3 ± 0.9	98.7 ± 3.7	9.37	11.6 *	25.0 ± 0.1	24.2 ± 0.4	2.56	0.8	1332.8 ± 15.6	1299.3 ± 60.6	0.29	33.5
10	13.0 ± 0.1	10.5 ± 0.4	34.73	2.5 *	118.1 ± 0.9	111.5 ± 2.5	6.01	6.6 *	24.8 ± 0.1	24.4 ± 0.3	1.07	0.4	1449.5 ± 15.3	1585.8 ± 41.8	9.40	−136.3 *
11	15.5 ± 0.1	12.3 ± 0.5	45.40	3.2 *	121.6 ± 0.9	110.4 ± 2.8	14.09	11.2 *	24.4 ± 0.1	23.9 ± 0.3	2.40	0.5	1379.8 ± 14.8	1610.6 ± 47.0	21.91	−230.8 *
12	17.7 ± 0.1	13.2 ± 0.4	113.59	4.5 *	124.7 ± 0.8	115.5 ± 2.5	12.76	9.2 *	24.5 ± 0.1	25.4 ± 0.3	8.42	−0.9 *	1375.4 ± 13.3	1492.7 ± 40.7	7.50	−117.3 *
13	19.4 ± 0.1	15.8 ± 0.5	53.84	3.6 *	121.6 ± 0.9	125.7 ± 2.8	1.90	−4.1	24.2 ± 0.1	23.9 ± 0.3	0.49	0.3	1339.6 ± 14.9	1415.4 ± 47.0	2.36	−75.8
14	20.2 ± 0.1	17.3 ± 0.3	60.05	2.9 *	121.9 ± 0.9	111.6 ± 2.1	20.0	10.3 *	24.8 ± 0.1	24.9 ± 0.3	0.02	−0.1	1402.9 ± 14.5	1329.9 ± 35.5	3.63	73.0
15	21.3 ± 0.2	18.4 ± 0.4	36.14	2.9 *	124.0 ± 1.3	121.6 ± 2.7	0.64	2.4	24.7 ± 0.2	24.7 ± 0.3	0.02	0.0	1451.8 ± 21.4	1466.5 ± 45.0	0.09	−14.7
	**Two-Factor ANOVA Model Results**															
**Age**	F(9, 4366) = 294.43, *p* < 0.01, η^2^ = 0.38				F(9, 4366) = 48.66, *p* < 0.01, η^2^ = 0.09				F(= 9, 4366) = 16.72, *p* < 0.01, η^2^ = 0.03				F(9, 4366) = 14.57, *p* < 0.01, η^2^ = 0.03			
**Stunted**	F(1, 4366) = 181.07, *p* < 0.01, η^2^ = 0.04				F(1, 4366) = 26.57, *p* < 0.01, η^2^ = 0.01				F(1, 4366) = 2.13, *p* = 0.14, η^2^ = 0.000				F(1, 4366) = 11.00, *p* < 0.01, η^2^ = 0.003			
**Age by Stunted**	F(9, 4366) = 3.36, *p* < 0.01, η^2^ = 0.01				F(9, 4366) = 2.99, *p* < 0.01, η^2^ = 0.01				F(9, 4366) = 2.48, *p* < 0.01, η^2^ = 0.01				F(9, 4366) = 3.56, *p* < 0.01, η^2^ = 0.01			

Note: * *p* < 0.05.

**Table 4:** Mean differences (Mean ± SD, F tests and Dif) and two-factor ANOVA model results for each physical fitness test between stunted and normal-growth boys.

Age (y)	Handgrip (kgf)				Standing Long Jump (cm)				Shuttle-Run (s)				12 min run (m)			
	Normal	Stunted	F	Dif	Normal	Stunted	F	Dif	Normal	Stunted	F	Dif	Normal	Stunted	F	Dif
6	7.5 ± 0.2	5.8 ± 0.7	5.33	1.7	94.7 ± 1.4	87.8 ± 4.2	2.47	6.9	25.3 ± 0.1	26.8 ± 0.4	10.63	−1.5 *	1120.3 ± 25.9	1101.8 ± 80.9	0.05	−18.5
7	8.5 ± 0.2	7.1 ± 0.7	3.37	1.4	105.6 ± 1.2	92.7 ± 4.2	8.67	12.9 *	24.9 ± 0.1	25.4 ± 0.4	1.05	−0.5	1295.5 ± 22.8	1503.0 ± 81.0	6.10	−207.7 *
8	10.1 ± 0.2	8.5 ± 0.6	7.32	1.6	112.5 ± 1.2	111.2 ± 3.2	0.13	1.3	24.2 ± 0.1	24.2 ± 0.3	0.00	0.0	1363.0 ± 22.9	1355.6 ± 62.0	0.01	7.4
9	12.1 ± 0.2	8.9 ± 0.8	17.12	3.2 *	116.4 ± 1.1	111.0 ± 4.4	1.40	5.4	23.5 ± 0.1	24.4 ± 0.5	3.45	−0.9 *	1418.7 ± 20.4	1666.7 ± 84.4	8.16	−248.0 *
10	13.6 ± 0.2	11.1 ± 0.6	18.57	2.5 *	123.6 ± 1.1	119.2 ± 3.2	1.73	4.4	23.1 ± 0.1	23.0 ± 0.3	0.02	0.1	1624.8 ± 21.1	1635.7 ± 61.1	0.03	−10.9
11	15.7 ± 0.2	13.0 ± 0.5	23.15	2.7 *	130.4 ± 1.1	124.1 ± 3.1	3.74	6.3	22.8 ± 0.1	22.3 ± 0.3	4.81	0.5	1658.1 ± 20.7	1863.5 ± 59.0	10.77	−205.4 *
12	18.8 ± 0.2	14.1 ± 0.5	71.80	4.7 *	131.9 ± 1.1	126.1 ± 3.0	3.37	5.8	22.8 ± 0.1	23.5 ± 0.3	3.81	−0.7 *	1641.0 ± 20.6	1731.9 ± 57.2	2.23	−90.9
13	23.2 ± 0.2	16.9 ± 0.4	176.27	6.3 *	137.9 ± 1.3	134.2 ± 2.4	1.83	3.7	22.4 ± 0.1	22.6 ± 0.3	0.64	−0.2	1623.2 ± 24.0	1667.4 ± 46.8	0.71	−44.2
14	28.4 ± 0.2	20.9 ± 0.4	262.57	7.5 *	151.8 ± 1.2	137.1 ± 2.4	30.20	14.7 *	22.0 ± 0.1	22.4 ± 0.2	2.87	−0.4	1793.6 ± 22.7	1756.7 ± 45.9	0.52	36.9
15	31.4 ± 0.3	25.8 ± 0.6	84.11	5.6 *	159.5 ± 1.4	150.3 ± 3.2	6.87	9.2 *	21.6 ± 0.2	22.3 ± 0.3	3.08	−0.7 *	1924.5 ± 27.6	1881.6 ± 61.9	0.40	42.9
	**Two-Factor ANOVA Model Results**															
**Age**	F(9, 3508) = 571.24, *p* < 0.01, η^2^ = 0.59				F(9, 3508) = 109.14, *p* < 0.01, η^2^ = 0.22				F(9, 3508) = 44.24, *p* < 0.01, η^2^ = 0.10				F(9, 3508) = 40.36, *p* < 0.01, η^2^ = 0.09			
**Stunted**	F(1, 3508) = 351.53, *p* < 0.01, η^2^ = 0.09				F(1, 3508) = 37.81, *p* < 0.01, η^2^ = 0.01				F(1, 3508) = 11.51, *p* < 0.01, η^2^ = 0.003				F(1, 3508) = 10.14, *p* < 0.01, η^2^ = 0.003			
**Age by stunted**	F(9, 3508) = 14.99, *p* < 0.01, η^2^ = 0.04				F(9, 3508) = 2.10, *p* < 0.01, η^2^ = 0.01				F(9, 3508) = 2.43, *p* < 0.01, η2 = 0.01				F(9, 3508) = 2.43, *p* < 0.01, η^2^ = 0.01			

Note: * *p* < 0.05.
